# A Comparison of 16S rRNA Profiles Through Slaughter in Australian Export Beef Abattoirs

**DOI:** 10.3389/fmicb.2019.02747

**Published:** 2019-11-29

**Authors:** Sanga Kang, Joshua Ravensdale, Ranil Coorey, Gary A. Dykes, Robert Barlow

**Affiliations:** ^1^School of Public Health, Curtin University, Bentley, WA, Australia; ^2^Agriculture and Food, Commonwealth Scientific and Industrial Research Organisation (CSIRO), Brisbane, QLD, Australia; ^3^School of Molecular and Life Sciences, Curtin University, Bentley, WA, Australia

**Keywords:** 16S rRNA sequencing, beef microbiota, microbial contamination, beef slaughter, beef contamination, beef supply chain

## Abstract

Microbial contamination of beef cattle carcases and subsequent cross-contamination during processing is inevitable and virtually impossible to prevent. The understanding of microbial contamination in the beef industry is currently limited to hypotheses based on traditional microbiological tools. Additionally, the complex structural and functional responses of beef cattle microbial communities to the fragmentation in the supply chain remain unknown. This study used 16S rRNA gene sequencing in combination with traditional microbiology to monitor and compare changes in the microbiota throughout slaughter in an integrated (abattoir A) and a fragmented (abattoir B) beef abattoir in Australia. Briefly, the primary difference between an integrated and a fragmented abattoir is that fragmented abattoirs receive cattle from multiple sources, whereas integrated abattoirs typically receive cattle that has been produced using the same production system and from a limited number of sources. The composition in the bacterial communities varied between the abattoirs, though the presence of the most predominant bacterial species within the microbiota at each abattoir was similar. Lactobacillales (2.4–56.2%) and Pseudomonadales (2.4–59.4%) most notably dominated hides, carcases, and the environment in abattoir B. In abattoir A, Bacteroidales (3.9–43.8%), Lactobacillales (0.0–61.9%), and Pseudomonadales (0.5–72.1%) fluctuated but generally shared the dominance over the rest. Combined results of total viable count (TVC) and 16S rRNA gene profiling indicated that an upward hide pulling system adopted by abattoir B may lead to increased transmission of hide contaminants to post-hide pull carcases. Abattoir B had 3.2 log_10_CFU/cm^2^ reduction from hide to carcase, where abattoir A had 4.5 log_10_CFU/cm^2^ reduction. The findings from this study indicated that common beef-associated microbiota exist in varying composition in Australian abattoirs, and 16S rRNA amplicon sequencing is a powerful tool to understand in-depth movement of microbial contaminants.

## Introduction

The general route of contamination involves fecally contaminated hides transferring fecal matter and contaminants onto carcases during slaughter and processing of the animals (McEvoy et al., 2000; Barkocy-Gallagher et al., 2003; Arthur et al., 2004; Bosilevac et al., 2005). Transfer of microbial contaminants from the hide to carcase is likely to occur during hide removal resulting in the contaminated carcases becoming a vector for transmission of microorganisms including pathogens to different cuts of beef throughout the supply chain (Fegan et al., 2009; Arthur et al., 2010; Chopyk et al., 2016). Such movement and prevalence of microbial contaminants including the regulatory important pathogens in the beef supply chain are well documented in the literature (Elder et al., 2000; Collis et al., 2004; Fegan et al., 2005, 2009; Antic et al., 2010; Barlow and Mellor, 2010; Svoboda et al., 2013; Stromberg et al., 2015).

Australian beef processing abattoirs may operate as an integrated or a fragmented supply chain. Currently, fragmented supply chains appear to be the dominant type of supply chain in the Australian beef industry (Australian Meat Processor Corporation, 2017). In an integrated supply chain, beef cattle are received by processors from the same suppliers using consistent production strategies resulting in herds of animals arriving with minor variation in traits. In a fragmented supply chain, the processors receive animals from multiple different producers, which may result in herds of cattle with variations in physical traits such as weight, feed type and breed, as well as physiological and stress levels (Australian Competition and Consumer Commission, 2016). Therefore, one could argue that higher fragmentation in the supply chain may contribute to increase variability in the microbial composition due to beef cattle arriving from multiple different sources potentially carrying diverse endogenous microbial populations.

The use of traditional microbiological techniques to analyze contamination of beef carcases in previous studies focused the understanding of contamination on easily culturable microorganisms. These techniques have assisted in understanding the general movement of microbial contaminants from feces to hides and ultimately to the carcases during beef slaughter (Bell, 1997; Fegan et al., 2005; Arthur et al., 2010; Pointon et al., 2012; Stromberg et al., 2015). The advent of high-throughput, next-generation sequencing (NGS) has enabled the understanding of the biodiversity in a given environment to be explored in much finer detail and complexity (Cao et al., 2017; Vollmers et al., 2017; Zaheer et al., 2018). It has been utilized to explore the microbiome in beef abattoirs in recent years and demonstrated that 16S rRNA sequencing acts as a viable tool to monitor sources of microbial contamination (De Filippis et al., 2013; Bakhtiary et al., 2016; Chopyk et al., 2016; Yang et al., 2017). Research in the Australian beef industry has typically focused on the general microbial contamination levels of chilled beef carcases and beef products, pathogen surveillance, and antimicrobial resistance (Sumner et al., 2003; Jordan et al., 2007; Fegan et al., 2009; Barlow and Mellor, 2010; Phillips et al., 2012; Barlow et al., 2015; Mellor et al., 2016). In the current literature, Chandry (2013, 2016) described the bacterial profiles in different sample types collected from an Australian export beef abattoir in two industry project reports using 16S rRNA amplicon-based approach. There are, however, no published articles on the use of amplicon-based studies in Australian red meat production systems.

This study was designed to map and compare the flow of bacterial profiles during slaughter in two Australian beef export abattoirs with a varying level of supply chain integration using 16S rRNA amplicon sequencing. Fragmentation in the supply chain is hypothesized to have an effect on microbial ecology present during slaughter by introducing variation in the microbial load entering the abattoir. This hypothesis is tested by comparing the analysis of 16S rRNA profiles between two Australian beef export abattoirs with varying integration in the supply chain. Additionally, the abattoirs are examined to provide an insight into recognizing parts of the slaughtering process that contribute the most to carcase contamination and understand the movement of contaminants with natural variations in the process.

## Materials and Methods

### Sample Collection and Experimental Design

Samples were collected from two abattoirs with an integrated and a fragmented supply chain in Australia. The integrated abattoir (A) employed downward hide pull (DHP) system, where the fragmented abattoir (B) used upward hide pull (UHP) system. Neither abattoir employed specific antimicrobial interventions and the locations and frequency of trimming was comparable between the two abattoirs. Each abattoir was each visited twice with a period of 3 months between the visits (between January and July 2018), and 90 samples were collected per visit. Abattoirs A and B had similar processing line speed at 80–100 carcases per hour. The samples collected consisted of 10 fecal samples, 15 hide samples prior to hide-pull, 15 carcase samples immediately after hide-pull, 15 carcase samples post-evisceration, 15 carcase samples immediately before chilling (pre-chill), and 20 environmental samples throughout the slaughter floor. For the environmental samples, 10 sites were chosen, and each site was periodically sampled twice. The samples were collected after sanitation and within an hour of slaughter commencement in the morning of the day. Each and every sampling visit was ensured to collect samples at this time of the slaughter day. The sampling sites were sequentially matched in a spatial order of slaughter process but were not identical between the abattoirs.

Large area sampling was used as previously described (Chandry, 2013). In brief, hide and carcase samples were collected aseptically by swabbing forequarter of the animals using large area (3,000 cm^2^) sampling technique. Environmental samples were obtained from swabbing an area approximately equivalent to 900 cm^2^. Fecal samples were collected from the internal content of freshly deposited fecal pats in holding pens before slaughter. Swabbing of carcase and environmental samples was performed using Whirl-Pak^®^ Speci-Sponge^®^ (Nasco, Fort Atkinson, Wisconsin) pre-moistened in 25 ml of sterile buffered peptone water (BPW; Oxoid, Basingstoke, UK). Fecal samples were collected in individual yellow capped plastic jars (Sarstedt, Numbrecht, Germany) using a sterile spoon. All samples were immediately placed on ice and transferred to the laboratory within 4 hours of collection. At the laboratory, all samples collected with a sponge had an additional 75 ml of BPW added prior to being stomached for 30 s at four strokes per second (Interscience, Saint Nom, France). Fecal samples (approx. 10 g) were diluted 1 in 10 with BPW and stomached for a minute. Following stomaching, aliquots from the samples were kept for 16S rRNA sequencing analysis at −80°C, and the remaining portion of the bacterial-BPW suspension was used for microbiological analysis.

### Microbiological Examination of Samples

Bacterial-BPW suspensions were used for total viable count (TVC). TVCs were obtained by plating 100 μl aliquots of serial 10-fold dilutions prepared in saline (Oxoid, Basingstoke, UK) onto tryptic soy agar (TSA; Oxoid, Basingstoke, UK) and incubated at 25°C for 72–96 h. Statistical analysis by *t* test comparing two means for continuous data was performed using GraphPad Prism version 8.0 for Windows, https://www.graphpad.com/.

### 16S rRNA Amplicon Sequencing Analysis

#### DNA Extraction

A 1 ml aliquot from hide and fecal samples were centrifuged to pellet the cells. For carcase and environmental samples, a 40 ml aliquot was used to pellet the cells. The pellets were washed two times using ultrapure H_2_O prior to DNA extraction using QIAamp PowerFecal DNA kit (Qiagen, Valencia, CA, USA) following the manufacturer’s instructions with a modified bead beating step. The bead beating procedure involved a total of 3 min of beating with a minute rest after the first and second minutes of beating. The bead tubes were then placed in heating blocks at 65°C for 10 min, and the bead beating was repeated.

#### Preparation of 16S rRNA Library

Extracted DNA was utilized to construct a sequencing library targeting the V4 region of 16S rRNA gene using a previously published protocol (Kozich et al., 2013). Briefly, an aliquot of 5 μl from each DNA template was amplified with dual-index primers *via* PCR, and the amplicons were manually normalized. Each template contained specific barcode sequences at the 5′ and 3′ of the PCR amplicon to allow stratification among each other in the pooled library on the Illumina sequencing platform. The concentration of the amplicons was estimated by visually comparing the intensity of the DNA band against the GeneRuler 100 bp Plus DNA Ladder (ThermoFisher, Scoresby, VIC, Australia) stained with ethidium bromide in 2% agarose gel under ultraviolet light. A final 16S amplicon concentration of 50 ng from each sample was combined to generate a pooled library. Additionally, ZymoBIOMICS™ microbial community DNA standard (Zymo Research, Irvine, CA, USA) was normalized and added to the library. After normalization, an aliquot of approximately 200 μl was used for purification *via* Agencourt AMPure XP magnetic beads (Beckman Coulter, Brea, CA, USA) using the manufacturer’s instruction. The purity and concentration of DNA were estimated pre- and post-purification using a NanoPhotometer^®^ (IMPLEN, UK) and Qubit^®^ 2.0 Fluorometer (Life technologies, CA, USA), respectively.

#### Sequencing Using Illumina MiSeq

Sequencing of the amplicons was conducted at University of New South Wales (UNSW) using the Illumina MiSeq platform (Illumina, San Diego, CA, USA) with a paired-end 300 base pair sequencing protocol. A pooled library (20 nM) and a PhiX control v3 (20 nM) (Illumina) were mixed with 0.2 N fresh NaOH and HT1 buffer (Illumina) to produce the final concentration at 12 pM each. The resulting library was mixed with the PhiX control v3 (5%, v/v) (Illumina) and 600 μl loaded on a MiSeq1 v2 (500 cycles) Reagent cartridge for sequencing. All sequencing procedures were monitored through the Illumina BaseSpace® website.

#### Bioinformatic Analysis

Both de-multiplexed R1 and R2 sequencing read (approximately 250 bp in length) files were acquired from the Illumina BaseSpace® website, and data processing was performed using the v1.40.5 MOTHUR pipeline (Schloss et al., 2009) following the MiSeq standard operating procedures (Kozich et al., 2013). Paired end reads were generated and clustered prior to assembly into Operational Taxonomic Unit (OTU) tables with 97% identity. Representative sequences from the SILVA 16S rRNA gene database (v132) were used to classify reads into the respective taxonomical level from domain to genus. Subsequent bacterial community structure and similarity were measured using the PRIMER-7 (version 7.0.13, Primer-E, Ivybridge, UK) software package. For analysis of the community data, Bray-Curtis similarity matrix and non-metric multidimensional scaling (nMDS) were used within PRIMER-7. For analysis of transition of microbial communities, average of relative abundance for OTUs for each sample type was calculated in Excel. OTUs that were less than 0.5% of the total 16S rRNA sequence reads were excluded from the list. The OTUs that were above 0.5% approximately represented 98% of the total within each sample group.

## Results

### The Presence of Microorganisms Throughout the Slaughter Floor and Abattoir Environment

**Figure 1:**
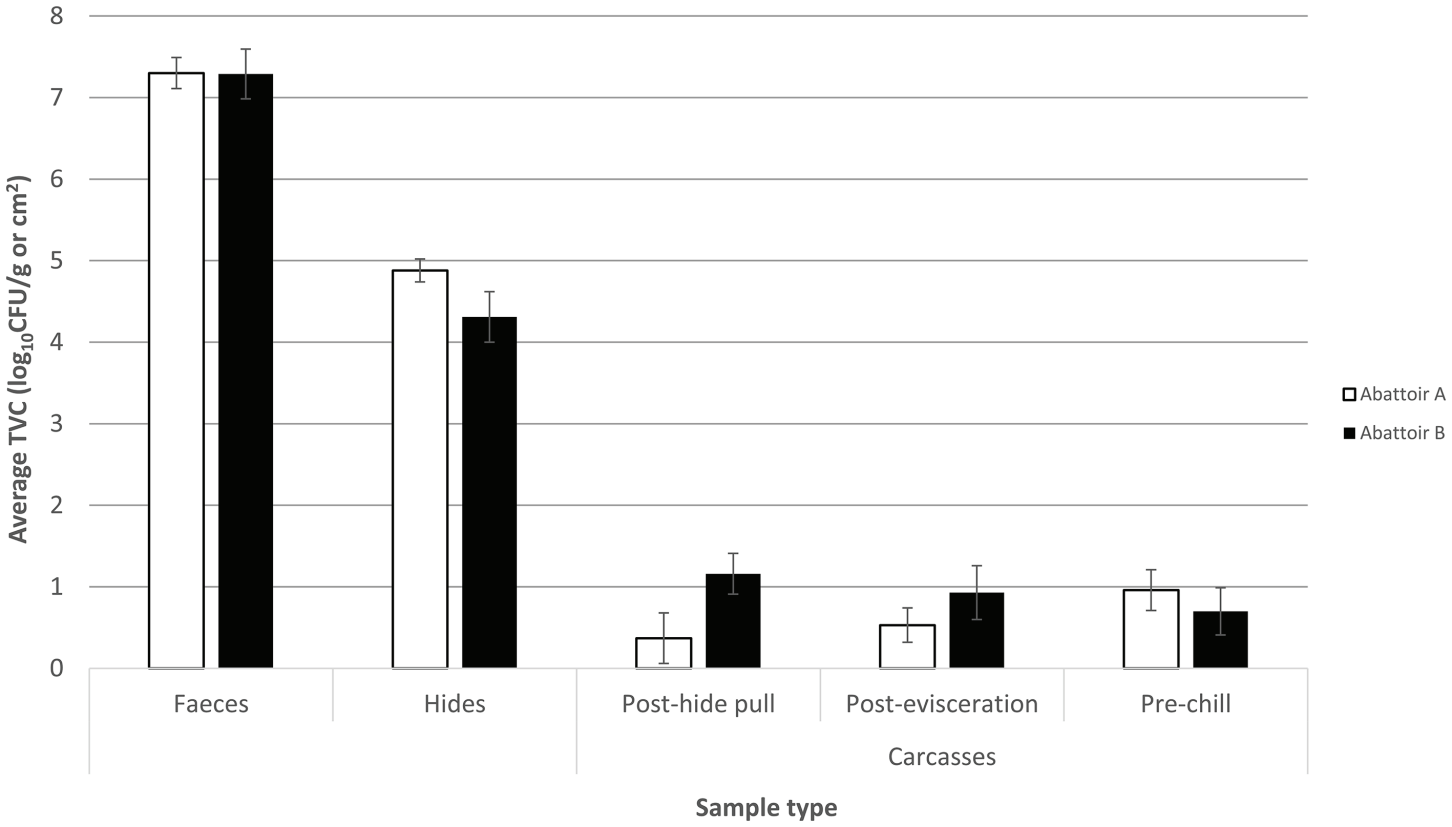
Mean total viable count (TVC) of fecal, hide, post-hide pull, post-evisceration, and pre-chill carcase samples collected from the slaughter floor of an integrated (abattoir A) and a fragmented (abattoir B) Australian beef supply chain. Mean TVCs of fecal samples are reported in log_10_CFU/g with the hide and carcase samples reported in log_10_CFU/cm^2^. Each column represents the mean of *X* samples with error bars representing the standard deviation from the mean.

Total viable counts (TVCs) of feces, hides, and carcases at three different stages (post-hide pull, post-evisceration, and pre-chill) were determined and are shown in Figure 1. The mean TVC was the highest in fecal samples for both abattoirs with a mean count of 7.3 log_10_CFU/g for the integrated (A) and fragmented (B) abattoirs. Mean TVC in hide samples was 4.9 and 4.3 log_10_CFU/cm^2^ in abattoirs A and B, respectively. Abattoir A removed the hides using a downward hide pull (DHP) system resulting in mean TVC in post-hide pull carcases of 0.4 log_10_CFU/cm^2^. On the other hand, abattoir B removed the hides utilizing an upward hide pull (UHP) system, and the mean TVC in hides samples was significantly higher (*p* < 0.05) than post-hide pull carcases samples from abattoir A at 1.2 log_10_CFU/cm^2^.

The counts of post-evisceration carcases from abattoir B were also significantly higher (*p* < 0.05) than abattoir A, while there was no statistical difference (*p* < 0.05) in mean TVC of pre-chill carcase samples between the abattoirs (Figure 1). Abattoirs A and B showed opposing trends of mean TVC in carcases as they progressed through slaughter after the hide was removed. In abattoir A, counts of the carcases significantly increased (*p* < 0.05) moving through slaughter shown by the mean TVC of 0.4 log_10_CFU/cm^2^ in post-hide pull carcases increasing to 1 log_10_CFU/cm^2^ in pre-chill carcases. By contrast, the counts of carcases significantly decreased (*p* < 0.05) through the slaughtering process in abattoir B from the mean TVC of 1.2 log_10_CFU/cm^2^ in post-hide pull carcases to 0.70 log_10_CFU/cm^2^ in pre-chill carcases.

**Figure 2:**
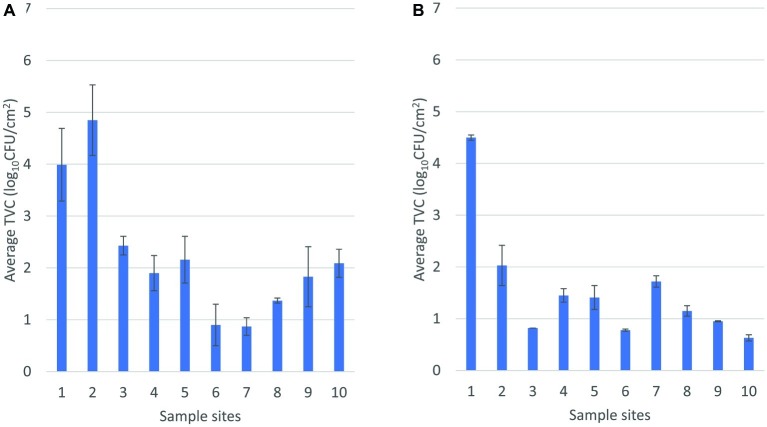
Mean total viable count of environmental samples sequentially arranged throughout the slaughter floor in abattoir A **(A)** and abattoir B **(B)**. In **(A)**, 1 and 2, pre-hide pull; 3, post-hide pull; 4, post-evisceration; 5, pre-scales; 6 and 7, post-scales; 8, pre-trim; 9, trimming; 10, chiller. In **(B)**, 1, pre-hide; 2 and 3, post-hide; 4, pre-evisceration; 5, post-evisceration; 6, pre-scales; 7, post-scales; 8 and 9, post-trim; 10, chiller entry.

Direct numerical comparison of TVC in environmental samples between the abattoirs cannot be drawn due to the fact that the sampling sites were not identical. There was a noticeable trend of higher counts in abattoir A from areas surrounding the hide removal station in comparison to the corresponding areas in abattoir B (1–3, Figure 2). The mean TVC in the environment between pre- and post-hide pull area was 3.8 and 2.5 log_10_CFU/cm^2^ in abattoirs A and B, respectively. Higher mean TVCs were observed toward the end of slaughter in abattoir A compared to abattoir B. Indeed, the overall trend for TVC in environmental samples from abattoir A was to increase with proximity to the chillers, whereas abattoir B demonstrated reductions in TVC through the processing line. There may be a number of factors for this disparity in counts (e.g., processing line design, speed of the rail, trimming technique, and aerosol control management), and the trend cannot accurately compare the realistic differences in counts at each stage. Nonetheless, the highlighting point is that the changes in TVC in the environment similarly reflected the trend of TVC observed in carcases throughout slaughter within each abattoir and that the trends in changes of TVC in abattoir A did not share common patterns with abattoir B.

### Changes in Composition of the Microbiota in Fecal, Hide, and Carcase Samples

**Figure 3:**
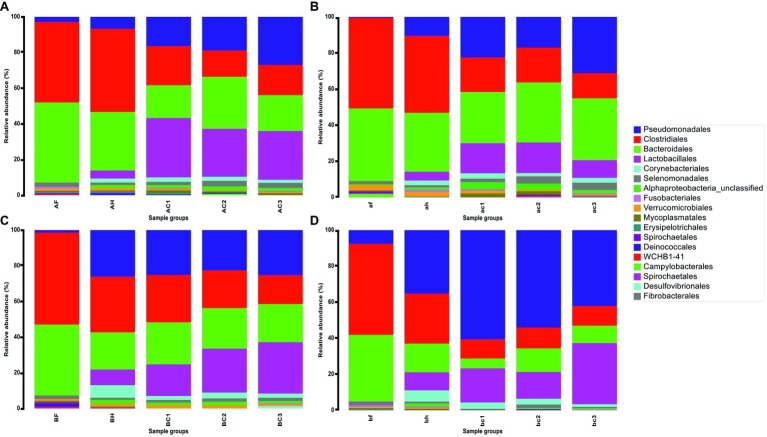
Relative abundance (%) of 18 OTUs at the Order taxonomy in fecal, hide, and carcase groups. **(A)** First visit to abattoir A; **(B)** second visit to abattoir A; **(C)** first visit to abattoir B; and **(D)** second visit to abattoir B. The groups are labeled in upper and lower cases for the first and second visits to the abattoir, respectively. There are three sample groups; fecal (F or f), hide (H or h), and carcase (C or c) groups. C1, C2, and C3 represent post-hide pull, post-evisceration, and pre-chill carcases, respectively.

A total of 12 million 16S rRNA gene sequence reads were obtained and were clustered into 122 operational taxonomic units (OTUs) at the order level. OTUs that were present at <0.5% of the total sequence reads within each group were excluded from the list for the following analysis. The 18 most abundant OTUs across all sample types represented at least 98% of the total sequence reads and were plotted to assess changes and transition in the diversity of the microbial community from feces in the holding pen to the carcases in the chiller. Relative abundance of OTUs in each sample was calculated, and the average value of relative abundance for each sample type is shown in Figure 3. It is important to highlight that relative abundance of OTUs hereafter is used or referred to as a measurement to describe the change in sequence reads within or between the groups.

Similarities in relative abundance of 18 OTUs and changes in community composition in hide and carcase samples between abattoirs A and B are shown in Figure 3. The hide sample groups in abattoir A were predominantly comprised of Clostridiales (A: 45.6% and B: 41.4%) and Bacteroidales (A: 32.1% and B: 31.6%), while the other OTUs remained below 10% (Figures 3A,B). In abattoir B, Clostridiales (C: 29.9% and D: 27.0%) and Bacteroidales (C: 19.9% and D: 15.5%) were also predominant, but an increase in the presence of Pseudomonadales (C: 25.0% and D: 34.3%) was a notable feature in the hide microbial community (Figures 3C,D). Additionally, a contrasting trend of Pseudomonadales between the abattoirs was observed with Pseudomonadales in carcase microflora from abattoir A generally increasing as slaughter progressed (A: 16.1–26.4% and B: 22.0–30.4%) but was either maintained or decreased in abattoir B as slaughter progressed (C: 24.3–24.5% and D: 59.4–41.5%). The opposite pattern occurred in the trend of changes in carcase Lactobacillales levels where it decreased in abattoir A (A: 32.6–26.7% and B: 16.3–9.5%) but increased in abattoir B (C: 17.0–27.9% and D: 18.5–33.4%).

An interesting trend was observed in the transition of microbial contaminants from the hide (BH) to the carcase groups (BC1, BC2, and BC3) in abattoir B, which was not observed in abattoir A. On the first visit, BH and post-hide pull carcase group (BC1) shared almost identical community structures. Lactobacillales was the only OTU that had more than 5% change in the relative abundance: 8.4–17.0% (Figure 3C). Relative abundance of Clostridiales and Corynebacteriales decreased by 4.3 and 4.5% from hides to post-hide pull carcases (29.9–25.6% and 6.7–2.2%), respectively, while the remaining OTUs altered between 0.3 and 2.9%. The changes in bacterial community composition from the hide to post-hide pull carcase in the second visit were more apparent in comparison. Pseudomonadales increased from 34.3% in hide to 52.3% in carcase, while Lactobacillales increased from 9.7 to 18.5% (Figure 3D). Bacteroidales and Clostridiales decreased 10.1 and 16.6%, respectively, and the relative abundance of remaining OTUs was altered by less than 5%.

### Differences in Bacterial Composition and Diversity Between All Sample Types

A curated list of all OTUs at the order level was utilized to generate non-metric multidimensional scaling (nMDS) plots. This ordination physically represents the similarity in composition of the bacterial community between all sample groups (feces, hides, carcases, and environmental) from different sites using the Bray-Curtis distance matrix. For this analysis, OTUs that were present at more than 0.5% of the total sequences in each sample group were selected and added to the list of OTUs. A final list of 27 OTUs that covered 97% of total sequence reads in each sample group was generated. Environmental samples provided a wider range of OTUs in comparison to the three groups above (feces, hides, and carcases). Here, the effect of fragmentation in the supply chain on the indigenous microflora between and within the abattoirs is shown by direct comparison of beta diversity (visualized by nMDS) and bacterial community structure. This subsequently allowed investigation of the relationship between the environment and the carcases during slaughter.

**Figure 4:**
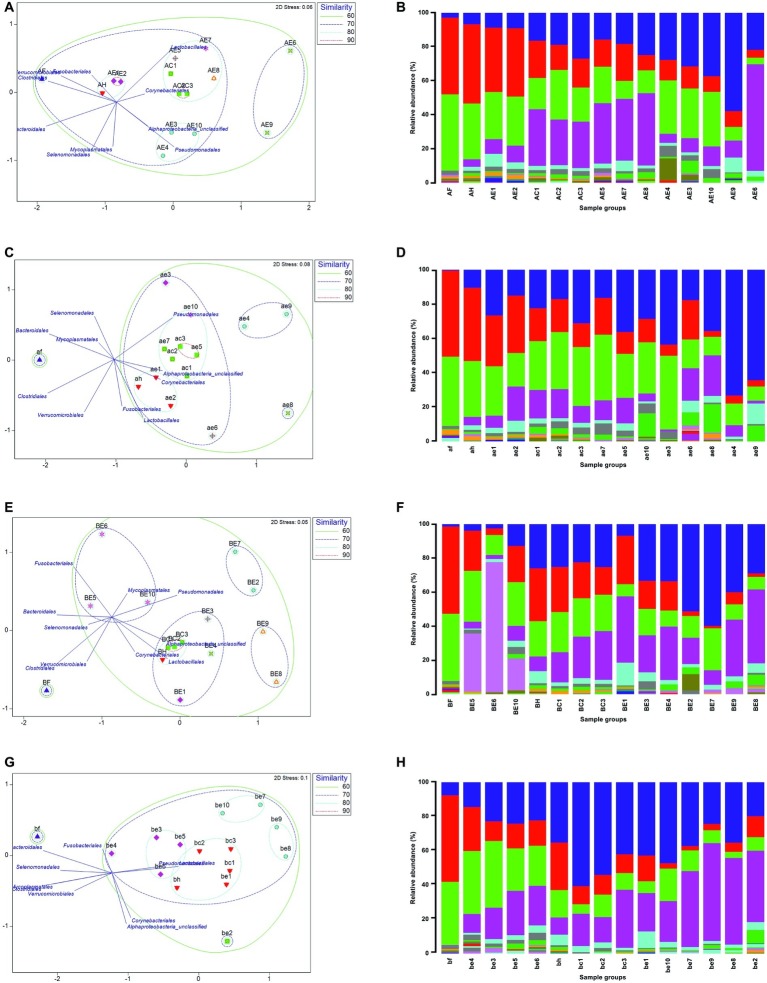
Non-metric multidimensional scaling (nMDS) ordination showing the similarity in bacterial community composition using S17 Bray-Curtis and overlay vectors of the top 10 OTUs (left) and relative abundance of 27 Order OTUs in all sample groups (right). First abattoir A visit is shown in **(A)** and **(B)** and second visit in **(C)** and **(D)**. First visit to abattoir B in **(E)** and **(F)**, and second visit in **(G)** and **(H)**. Physical distance between each group in the ordination indicates the similarity of the two or multiple groups. Displacement of the OTUs in the same ordination represents the distribution of each OTU. Fecal (F or f), hide (H or h), carcase (C or c), and environmental (E or e) groups are labeled in upper and lower cases for the first and second visits for each abattoir, respectively.

Lactobacillales in both visits to abattoirs A and B were predominantly present in the carcase groups and a number of common environmental sites as shown in Figure 4. Pseudomonadales were predominantly distributed in the environment in the first visit to abattoir B and in the carcases as well as in the environment in the second visit (Figures 4E,G). In contrast to abattoir A, Corynebacteriales and Alphaproteobacteria_unclassified were predominantly present in hides, carcases, and environmental sites. Intriguingly, three environmental sites (BE4, BE9, and BE10) from the first abattoir B visit were the only sample groups to have Fusobacteriales contributing a high proportion within the communities at 36.5, 14.5, and 57.2%, respectively (Figure 4C).

In abattoir A, the fecal groups shared 66% (A) and 59% (B) similarity with the composition of bacterial communities in the other sample groups (Figures 4A,B). The hide group was placed in the closest proximity to the fecal group on nMDS in both visits indicating that the hide group shared the most similar microbiological profiles with the fecal group. In abattoir B, the fecal group shared less similar community composition with the rest of the samples in comparison to abattoir A (C: 54% and D: 56%, Figure 4). Some consistency between the sample groups was observed across abattoir A visits, where the environmental sites 1 and 2 were more than 80% similar to the hide groups (Figures 4A,B). In the first abattoir A visit, all three carcase groups were approximately 70% similar to the composition of the hide group and were more than 80% similar to environmental sites 5, 7, and 8 (Figure 4A). On the second visit, the hide group shared at least 80% similarity in the bacterial community composition with the carcase groups in addition to environmental sites 5 and 7 (Figure 4B). In abattoir B, the hide groups were found in the closest proximity to the carcase groups in nMDS during both visits sharing more than 80% similarity in community composition (Figures 4C,D). Interestingly, all sample groups excluding the fecal group were at least 70% similar on the second visit to abattoir B meaning that three environmental sites (1, 3, and 5) were consistently found to be more than 70% similar (75–76%) to the carcase groups in abattoir B (Figure 4D).

## Discussion

This was the first study to utilize 16S rRNA sequence-based in a combined effort with traditional microbiology to monitor the flow of bacterial communities and compare the microflora between two Australian beef abattoirs with a different supply chain. Chandry et al. (2016) carried out studies in Australia using 16S rRNA amplicon sequencing and traditional methods where the group tracked and collected samples from matching hides and carcases in addition to collection of airborne bacteria at three different sites near the sampling stations. The study found that a substantial amount of airborne microflora derived from the hide and the aerosols played a role in contamination of the carcases (Madden et al., 2004; Burfoot et al., 2006; Cenci-Goga et al., 2007). There are other studies that have used NGS techniques to investigate sources of contamination in beef processing abattoirs (Chandry, 2013, 2016; De Filippis et al., 2013; Hultman et al., 2015). However, none of these studies have monitored flow and transition of microbial populations between the animals the abattoir environment throughout beef processing. Therefore, this study is the first of its kind and is aimed at understanding the flow of bacterial communities through the first stage of beef processing with an emphasis on the level of supply chain integration.

The combined results of bacterial community transition and composition similarities demonstrated that the movement of contaminants in abattoir B conformed to the traditional pathway of contamination more than abattoir A, i.e., feces to hides to carcases (Barkocy-Gallagher et al., 2003; Collis et al., 2004; Fegan et al., 2005; Arthur et al., 2010). These abattoirs harbored similar groups of beef-associated microbes such as Pseudomonadales, Lactobacillales, Corynebacteriales, Clostridiales, and Bacteroidales despite abattoir B receiving herds of beef cattle from multiple producers with varying traits as opposed to continuous supply of cattle with comparably consistent production traits (Habimana et al., 2010; De Filippis et al., 2013; Zaheer et al., 2017). However, the microbiological differences between the abattoirs were evident in the prevalence of these OTUs within the bacterial community. For instance, the hide samples from abattoir B had higher level of Pseudomonadales than abattoir A. This may have contributed to higher dominance of Pseudomonadales in carcase samples from abattoir B due to the close relationship that the hide group had with the carcase groups. Pseudomonadales and Lactobacillales were consistently found to be one of the predominant OTUs in carcase, hide, and environmental samples in abattoir B compared to abattoir A. It was not surprising to find these OTUs at such high proportion because of multiple beef spoilage bacteria such as *Pseudomonas* spp., *Lactobacillus* spp., and *Leuconostoc* spp. belong to the OTUs and that Pseudomonadales are ubiquitous soil bacteria that can spread in the processing environment (Ercolini et al., 2006; Koutsoumanis et al., 2006; Nychas et al., 2008).

The two abattoirs utilized different hide pulling systems with abattoir A employing a DHP system in their chain, whereas abattoir B has an UHP system. Carcases at both abattoirs were suspended on a single processing line by the hind limbs. DHP pulls the hide down from the hind leg to the neck after initial opening near the rump region, and UHP removes the hide up from the shoulder to the hind leg. The general consensus within the meat industry is that UHP leads to increased microbial load on the carcasses in comparison to DHP. UHP provides an opportunity for microorganisms to attach to otherwise sterile muscle tissue as the hide is vigorously plucked over the carcase. There is a lack of published data regarding this aspect, but different studies have shown that hide contamination generally is transferred to the carcases during processing (Bell, 1997; Svoboda et al., 2013; Chopyk et al., 2016). A study by Kennedy et al. (2014) investigated the microbial effect of the two hide pulling systems using TVC and found that the overall contamination of the carcase forequarter was not substantially affected by changing the direction of hide pull. Contamination increased in the flank and chuck after UHP and the shin and brisket after DHP leading to different products being contaminated by the change in the direction of hide pull. Implementation of hygienic hide removal practices (e.g., washing hands and knives between hide/carcase contact) were concluded as a more important factor for preventing overall forequarter contamination than the hide pulling method (Kennedy et al., 2014). This study produced an opposite outcome to the previous study and suggested that UHP system may induce increased microbial contamination in the forequarter of de-hided carcases in comparison to DHP system at least by half a log per cm^2^ on average. Here, the analysis of 16S rRNA profiles revealed that there is a profound and stronger microbial community similarity between the carcase and the hide groups in abattoir B (with UHP) than A (with DHP). Some studies have found that hindquarter of beef carcases harbored higher concentration of microbes (Charlebois et al., 1991; Gill et al., 1998; Phillips et al., 2012), meaning that greater differences may have been detected if sampling of hindquarters was conducted as part of this study. It is possible that airborne contamination in such a dynamic working vicinity could be playing a part and it would be beneficial to consolidate the findings from this study (Madden et al., 2004; Burfoot et al., 2006; Cenci-Goga et al., 2007; Chandry, 2016).

It is clear that carcases in abattoir A accumulated more bacteria on the forequarter of the carcases as they moved through slaughter than abattoir B. Such a trend of TVC on carcases throughout slaughter in abattoir B suggests that the abattoir is less efficient at minimizing the transfer of microbial contaminants from hide to carcase but may have counteractive management practices downstream to reduce the carcase bacterial load before chilling. Abattoir B was able to simultaneously reduce and maintain the level of microorganisms in the environment throughout slaughter after the hide pulling station. Analysis of 16S gene amplicons indicated that the microbes in the slaughter environment are likely to contribute to the carcase microflora. There is an ecological interaction between the bacterial communities in the carcase and the environment, but it is implausible to conclude that the microbes from the sampled environmental sites were the only contributor to the change of the carcase microflora in abattoir A or B (Kim and Yim, 2017; Yang et al., 2017). With that in mind, comparably lower levels of TVC in the environment in abattoir B may have contributed to progressive lowering of the carcase count in abattoir B. By contrast, removal of the hide in abattoir A leads to less contamination of the carcase than in abattoir B. However, the abattoir appeared to be less efficient at maintaining lower TVC in carcases and the environment throughout slaughter.

## Conclusion

The results from this study demonstrated that common meat-associated microorganisms are found throughout slaughter regardless of the level of integration in the supply chain of beef cattle. Microbiological differences were observed in the composition of the bacterial communities, especially in the environment, and the relationships between the different sample types were characteristic to individual abattoirs. The integrated abattoir showed efficient control of microbial contamination at the hide puller but was less efficient at controlling contamination as the carcases moved through the remainder of the slaughter process. By contrast, contamination of carcases in the fragmented abattoir conformed more to the traditional route of contamination (from feces to hides then to carcases), and it is likely that the direction of hide pull in abattoir B enhances the transfer of bacteria from hides to carcases. This study demonstrated that 16S rRNA amplicon-based analysis can be a powerful tool for understanding microbial ecology and specific interactions of microbial contaminants in commercial beef processing settings that may ultimately assist in controlling contamination events during slaughter. In a broad sense, 16S rRNA gene sequencing can potentially be used to manipulate or increase the presence of favored non-harmful cohort of bacteria or identify bioindicator/s for rapidly determining the microbiological quality of carcases. It is important to highlight that the microbiome at the end of slaughter is likely to be additionally affected as the carcases are processed further within a boning room. Further studies investigating the complete slaughter process including chilling and boning would facilitate greater understanding of the role of microbial ecology in the beef industry.

## Data Availability Statement

The raw 16S rRNA sequences generated for this study can be found in the European Nucleotide Archive (https://www.ebi.ac.uk/ena) under accession number PRJEB34187.

## Author Contributions

SK performed the experiments, analyzed the data and interpreted the results with guidance from RB. SK and RB engaged with the appropriate members of the industry and collected the samples. JR provided some assistance in molecular investigation of bacterial diversity. SK wrote the manuscript with contributions from RB, JR, RC, and GD.

### Conflict of Interest

The authors declare that the research was conducted in the absence of any commercial or financial relationships that could be construed as a potential conflict of interest.
